# *Beauveria bassiana* ERL836 and JEF-007 with similar virulence show different gene expression when interacting with cuticles of western flower thrips, *Frankniella occidentalis*

**DOI:** 10.1186/s12864-020-07253-y

**Published:** 2020-11-27

**Authors:** Sihyeon Kim, Jong Cheol Kim, Se Jin Lee, Mi Rong Lee, So Eun Park, Dongwei Li, Sehyeon Baek, Tae Young Shin, Jae Su Kim

**Affiliations:** 1 Department of Agricultural Biology, Jeonbuk National University, Jeonju, 54596 South Korea; 2 Department of Microbiology and Cell Science, University of Florida, Gainesville, FL 32611-0700 USA; 3 Department of Agricultural Convergence Technology, Jeonbuk National University, Jeonju, 54596 South Korea

**Keywords:** *Beauveria bassiana*, Western flower thrips, Genome, Transcription, Cytochrome P450

## Abstract

**Background:**

Insect-killing fungal species, *Beauveria bassiana*, is as an environment-friendly pest management tool, and many isolates are on the track of industrialization. However, some of *B. bassiana* isolates show similar morphology and virulence against insect pests, and so it is hard to differentiate them. Herein we used two patented isolates, ERL836 and JEF-007, and investigated their virulence against western flower thrips, *Frankliniella occidentalis*, and further analyzed genome structures and transcriptional responses when interacting with cuticles of thrips to see possible differences on the initial step of fungal infection.

**Results:**

The two isolates showed no significant differences in fungal growth, conidial production, and virulence against thrips, and they were structurally similar in genome. But, in transcription level, ERL836 appeared to infect thrips easily, while JEF-007 appeared to have more difficulty. In the GO analysis of ERL836 DEGs (differentially expressed genes), the number of up-regulated genes was much larger than that of down-regulated genes, when compared to JEF-007 DEGs (more genes down-regulated). Interestingly, in the enrichment analysis using shared DEGs between two infecting isolates, plasma membrane-mediated transporter activity and fatty acid degradation pathway including cytochrome P450 were more active in infecting ERL836.

**Conclusion:**

The two *B. bassiana* isolates had similar morphology and virulence as well as genome structure, but in transcription level they differently interacted with the cuticle of western flower thrips. This comparative approach using shared DEG analysis could be easily applied to characterize the difference of the two *B. bassiana* isolates, JEF-007 and ERL836.

**Supplementary Information:**

The online version contains supplementary material available at 10.1186/s12864-020-07253-y.

## Background

*Beauveria bassiana* has received great interest from both academia and industry because of its great potential as an alternative environment-friendly pest management tool to substitute synthetic pesticides that can damage the environment and cause insect resistance. Popularly available *B. bassiana* products are BotaniGard® (isolate name: GHA), Naturalis-L® (ATCC74040), Chongchaesak® (ERL836), Broadband® (PPRI 5339), and BioCeres® (ANT-03) and they are used to control agricultural insect pests such as moths, beetles, whitefly, aphid, mite, and thrips among others [1]. Particularly *B. bassiana* ERL836 GR has been developed by LG-Chemical-affiliated FarmHannong and successfully launched and sold out in a local market. Insect resistance to pesticides may not be an issue when using these fungal pathogens for pest control because this fungal group uses complicated mechanical hyphal penetration and enzymatic degradation to kill the host. This mode of fungal action is completely different from that of chemicals targeting synapses or energy metabolism. Although other entomopathogenic fungi such as *Metarhizium*, *Cordyceps* (previously *Isaria* or *Paecilomyces*), *Akanthomyces* (previously *Lecanicillium* and *Verticillium*) are also being investigated as pest management tools, *B. bassiana* has several advantages over these other fungi in terms of field application, mass production, and long-term storage.

Numerous *B. bassiana* isolates have been collected form natural environments and have been transferred to fungal libraries around the world. USDA-ARS manages a huge entomopathogenic fungal library and support academic research worldwide. Japan and China have big fungal libraries and Brazil and European countries also have entomopathogenic fungal libraries. Industrially important isolates from these libraries are placed in the research and development track for development as commercial or government-supported products. Very few isolates successfully pass all steps of industrialization to be launched in the biopesticide market. Nevertheless, the number of *B. bassiana* isolates being commercially developed is increasing. An important intellectual property (IP) issue is how to scientifically determine whether a particular *B. bassiana* isolate is different from another isolate. Attempts have been made to use phylogenetic analysis based on one or a couple of housekeeping genes, but this approach is not always adequate. As a prerequisite for intellectual property in South Korea, such as a patent for a particular isolate, clear experimental data that describes the uniqueness of an isolate needs to be submitted; this type of data could be used to resolve possible isolate-mediated conflicts in the biopesticide industry.

As introduced above, phylogenetic approaches have been mainly used to determine the evolutionary origins of isolates and their genetic relationships. Rehner and Buckley investigated the correspondence between *Beauveria* and *Cordyceps* using EF1-α and ITS phylogenies [2]. Glare et al. analyzed rDNA to determine phylogenetic relationships among 26 *Beauveria* isolates; more specifically, they used sequence information from the 3′ end of 16 s rDNA across ITS 1, 5.8 s rDNA, and ITS 2 to the 5′ end of 28 s rDNA [3]. In phylogenies, three nuclear genes encoding elongation factor 1-α (TEF1), RNA polymerase II largest subunit (RPB1), and RNA polymerase II second largest subunit (RPB2) were used by Rehner and colleagues [4]. Furthermore, a new species, *B. lii*, was found by four-locus based phylogenetic analysis [5]. However, these previous phylogenetic analyses were based on one or a couple of house-keeping genes, and could not be easily used to suggest that one isolate is different from another isolate in *B. bassiana* under IP issues.

Although fungal phylogenetic analysis can be used to suggest the uniqueness of one isolate in patent submission and acquisition, but the question is how many genes should be analyzed for this purpose. Whole genome sequencing (WGS) can provide the sequences of the entire genome of an organism and to compare the roles of genes or the diversity of genes in the isolates of same species of fungi. WGS of 10 *B. bassiana* isolates have been performed to date. ARSEF2860 (33.70 Mb, ADAH00000000.1) was the first WGS of *B. bassiana* and was obtained using Roche 454 system and Illumina paired-end sequencing in 2012 [6]. Genomes of other *B. bassiana* isolates, such as D1–5 (36.69 Mb, ANFO00000000) in 2014, ARSEF1520 (36.97 Mb, JTCW01000000), ARSEF 2597 (38.83 Mb, JTCX00000000), ARSEF8028 (35.02 Mb, JRHA00000000), ARSEF5078 (34.45 Mb, JTCZ00000000), ARSEF4305 (34.77 Mb, JTCY00000000) in 2016, BCC2660 (34.56 Mb, MWYT00000000) in 2017, and JEF-007 (36.54 Mb, MRVG00000000 from our previous work) and Bv062 (34.84 Mb, MAQY00000000) in 2018 have been generated. Whole genome sequences of D1–05, ARSEF1520, ARSEF 2597, ARSEF5078, and ARSEF4305 were obtained by Illumina sequencing.

To more deeply understand fungal mode of action, transcriptome analyses have been conducted, and this approach could be used to detect differences in patterns of gene expression among isolates of *B. bassiana*. De novo sequencing of non-model organisms is the most widely used strategy for transcriptomic profiling [7–9]. Some studies have investigated the interaction between *B. bassiana* and host insects at the molecular level. Transcriptome analysis of the initial phases of *B. bassiana* infection of the coffee berry borer, *Hypothenemus hampei*, was conducted [10]. Gene expression in silverleaf whitefly, *Bemisia tabaci*, in response to the infection by *B. bassiana* has also been analyzed [11]. Immunity-related gene expression in the Asian corn borer, *Ostrinia furnacalis*, when infected by *B. bassiana* was studied using an RNA-seq approach [12, 13]. Infection of *B. bassiana* to bean bug, *Riptortus pedestris* has also been investigated [14], as has the resistance and susceptibility of two silkworm species to *B. bassiana* infection [15]. Many virulence-related genes were found to be up-regulated in *B. bassiana* when infecting *Anopheles stephensi* mosquito [16]. Chen et al. studied the molecular mechanisms of *B. bassiana* infection to wax moth, *Galleria mellonella* [17]. Recently, in our laboratory Lee et al. performed transcriptome analysis of the bean bug in response to infection by *B. bassiana* [18]. In 2017, Wang et al. conducted a study to evaluate transcriptomic differences in two *B. bassiana* isolates when infecting silkworms [19].

Of the insects to be managed, thrips receives many attentions because of its high resistance to chemicals and cryptic behaviors. Transcriptome analyses of thrips infected with various entomopathogens have been conducted, but most studies have focused on gene expression of thrips, rather than fungal genes. Differentially expressed genes in western flower thrips, *Frankliniella occidentalis*, in response to tomato spotted wilt virus infection were analyzed by RNA-seq [20–23]. Zhang and colleagues identified a total of 36,339 thrips unigenes, and among them, 278 genes were involved in insecticide resistance [20]. Similarly, molecular responses of melon thrips, *Thrips palmi*, to capsicum chlorosis virus infection were analyzed by RNA-seq [24].

In this study, two patented *B. bassiana* isolates, JEF-007 (Patent No: 10–1666968, South Korea) and ERL836 (Patent No:10–1974265, South Korea; commercialized in a local market) were used and the following biological features were compared: hyphal growth, conidial production and virulence against thrips. Secondly, the genomes of the two isolates were compared. The whole genome of ERL836 (GenBank accession: PPTI00000000) was sequenced using PacBio technology and the genome sequence data for JEF-007 (GenBank accession: MRVG00000000) was obtained from our previous work [1]. Lastly the gene expression patterns of ERL836 and JEF-007 when interacting with cuticles of western flower thrips, *F. occidentalis* as the initial step of fungal infection, were analyzed using an RNA-seq technology.

## Results

### Fungal morphological growth

**Fig. 1:**
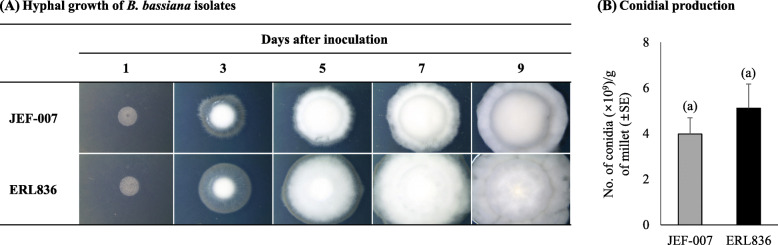
Hyphal growth (**a**) and conidial production (**b**) of *B. bassiana* ERL836 and JEF-007. A 2 μl of conidial suspension (1 × 10^7^ conidia/ml) was dropped on the middle of a ¼ SDA plate and kept in a 25 °C incubator for 9 days. Conidial productivity was evaluated using millet-based solid culture in polyethylene bags. A 1 ml of fungal conidial suspension (1 × 10^7^ conidia/mL) was inoculated to the bag including 200 g millet and incubated at 25 ± 2 °C for 10 days. Means followed by the same letter are not significantly different (*p* > 0.05)

The two isolates showed similar hyphal growth and conidial production (Fig. 1). The two isolates grew similarly on the 1/4SDA medium with white hyphal mass and conidia (Fig. 1a). On the millet-based solid culture, JEF-007 and ERL836 produced 3.98 ± 0.71 × 10^9^ conidia g^− 1^ and 5.12 ± 1.06 × 10^9^ conidia g^− 1^, respectively and no significant difference of conidial production was observed (F_*1,12*_ = 1.7, *p* = 0.783) (Fig. 1b).

### Virulence against thrips

**Fig. 2:**
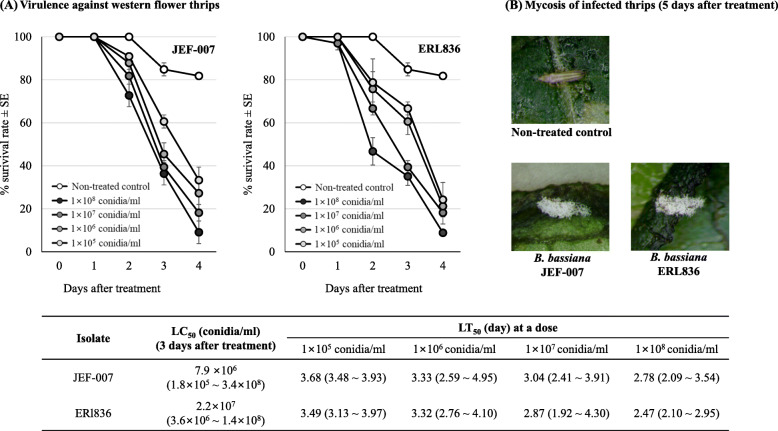
Insecticidal activity of *B. bassiana* ERL836 and JEF-007 against adults of western flower thrips in laboratory conditions (**a**) and mycosis of the infected thrips in 5 days (**b**). Virulence assays against adults of western flower thrips were conducted in laboratory conditions. Conidial suspensions were adjusted to 1 × 10^5^, 1 × 10^6^, 1 × 10^7^, and 1 × 10^8^ conidia ml^− 1^ and a 1-ml aliquot of conidial suspension was sprayed on a cucumber leaf disc in a Petri dish and ten two-day-old thrips adults were transferred to the fungus-treated dish (11 adults/dish). LC_50_ values of the two isolates were calculated using day-3 data, which was followed by LT_50_ data. Cadavers (mycosis) were observed 5 days after treatments. Means with the same letters are not significantly different (*p* > 0.05)

In the bioassay against western flower thrips, JEF-007 and ERL836 showed similarly high virulence against the adults under laboratory conditions (Fig. 2). When the adults were sprayed with the two isolates, the treated thrips showed fungus dose-dependent response and no significant mortality between the two isolate treatments. In 3 days after treatment, LC_50_ of JEF-007 was 7.9 × 10^6^ (1.8 × 10^5^ ~ 3.4 × 10^8^) conidia ml^− 1^ (*R*^*2*^ = 0.881) and LC_50_ of ERL836 was 2.2 × 10^7^ (3.6 × 10^6^ ~ 1.4 × 10^8^) conidia ml^− 1^ (*R*^*2*^ = 0.916) (Fig. 2a). The lethal time 50 (LT_50_) of the two isolates were similar in each conidial concentration. At 1 × 10^7^ conidia ml^− 1^, the LT_50_ of JEF-007 was 3.04 (2.41 ~ 3.91) days and that of ERL836 was 2.87 (1.92 ~ 4.30). Similar hyphal growth and sporulation on the insect cadavers (mycosis) were observed in the two fungal isolates-treated adults in 5 days after the treatment (Fig. 2b).

### Genome features of two B. bassiana isolates

From the de novo sequencing of the two isolates (Supplementary Table S1), the genome sizes of *B. bassiana* JEF-007 and ERL836 were similar to each other; 36.5 Mb of JEF-007 and 35.5 Mb of ERL836 (Table 1). These genome sizes were similar to the previously analyzed *B. bassiana* ARSEF2860 (33.7 Mb). Most genomic features of the three *B. bassiana* isolates were not significantly different, except for different scaffold numbers due to different sequencing platforms (ARSEF2860: 242, ERL836 and JEF-007: 15 and 39, respectively). The G + C content of the three isolates was approximately 48 ~ 52%, and the numbers of protein-coding genes were 10,631 (ERL836), 10,857 (JEF-007) and 10,366 (ARSEF2860).

**Table 1:** Genomic characterization of *B. bassiana* ERL836 and JEF-007

Genome features	*Beauveria. bassiana*		
	ERL836^**a**^	JEF-007^**a**^	ARSEF2860
Size (Mb)	**35.5**	**36.5**	33.7
Coverage (fold)	**108.1X**	**105.1X**	76.6X
Scaffold No. (> 1 kb)	**15**	**39**	237
Scaffold N50 (Mb)	**3.99**	**3.12**	0.73
% G + C content	**49**	**48**	51.5
% G + C in coding gene	**55.5**	**57.1**	56.6
% Repeat rate	**1.59**	**1.71**	2.03
Protein-coding genes	**10,631**	**10,857**	10,366
Protein families (protein no.)	**5381 (7788)**	**1284 (4282)**	3002 (7283)
Gene density (gene / Mb)	**299**	**297**	308
Exons per gene	**2.6**	**2.3**	2.7
% Secreted proteins	**19.5**	**18.9**	18.2
tRNA	**152**	**140**	113
NCBI accession	**PPTI00000000**	**MRVG00000000**	ADAH00000000

^a^ Whole genomes of the two isolates (*B. bassiana* ERL836 and JEF-007) were sequenced by Pac-Bio RSII technology and compared with previously sequenced *B. bassiana* ARSEF2860

### Comparison of genome structure of two *B. bassiana* isolates

**Fig. 3:**
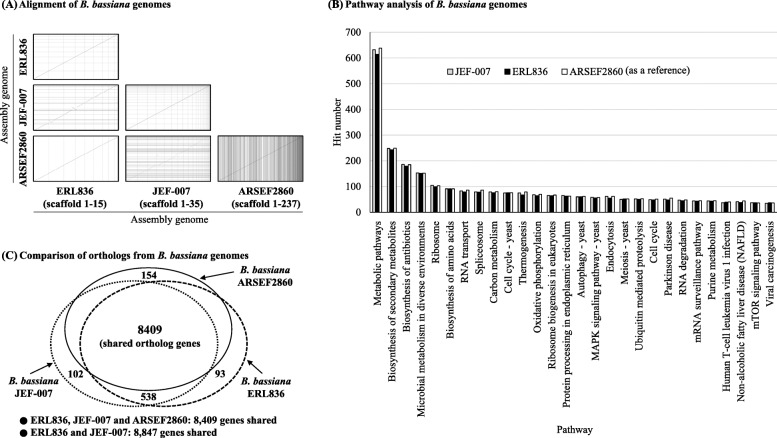
Comparative genome alignment (**a**), pathway analysis (**b**) and ortholog analysis (**c**) of *B. bassiana* ERL836 and JEF-007. In the alignment and pathway & ortholog analyses, *B. bassiana* ARSEF2860 was used as a reference genome for comparison. Alignments of ERL836 with JEF-007, ERL836 with ARSEF2860, and JEF-007 with ARSEF2860 were conducted using Assemblytics (http://assemblytics.com/). KEGG orthology was used to analyze pathways of three *B. bassiana* isolates (http://www.genome.jp/tools/kaas/). Identification of orthologous genes was conducted by bi-directional best hit (BBH)

No big significant differences in genome structure were observed between the two *B. bassiana* genomes (Fig. 3a). When ERL836 genome was aligned with JEF-007 genome, although there were some minor differences, but mostly genome structures (synteny) were similar to each other. When the genome of ERL836 was aligned to that of ARSEF2860 as a reference genome, two genome structures were similar, although some minor parts of the middle of the ARSEF2860 genome did not match the ERL836 genome. Additionally, when the three *B. bassiana* isolates were subjected to KEEG pathway analysis, no significant differences were detected (Fig. 3b). Metabolic pathway (9.7%), biosynthesis of secondary metabolites (3.9%) and biosynthesis of antibiotics (2.8%) were commonly major pathways in the three isolates. When orthologs of the two *B. bassiana* isolates were analyzed, ERL836 shared 8847 genes with JEF-007 and the two isolates shared 8409 genes with ARSEF2860 as a reference (Fig. 3c). In the three *B. bassiana* isolates, the percentages of the unique gene of each isolate were 0.19 ~ 0.35% of the total shared genes and the numbers are quite small.

### Comparative DEGs of two cuticle-interacting B. bassiana isolates

**Fig. 4:**
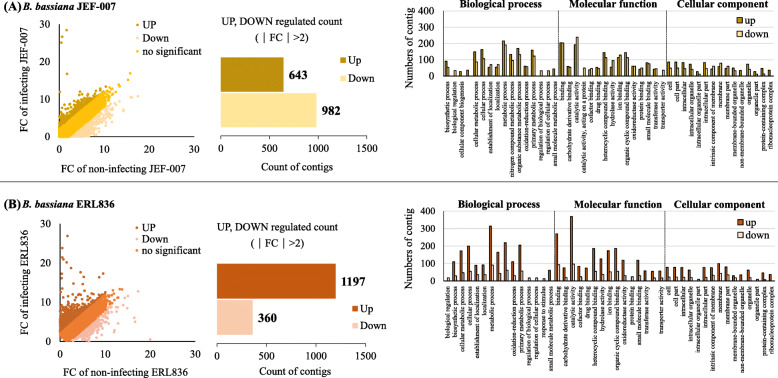
Differentially expressed genes (DEG) of *B. bassiana* ERL836 (**a**) and JEF-007 (**b**) when interacting with cuticle of western flower thrips. Thrips were exposed to 3-day old fungus-cultured plates for 3 days. Infected thrips were harvested using a surfactant solution and thrips bodies were excluded by filtering and subjected to RNA extraction. As a non-infecting control, 6-day cultured fungus was used for RNA extraction. In each isolate, the numbers of up- and down-regulated contigs (|fold change| > 2) were analyzed and GO analyses of DEGs were conducted using Blast2Go program

*B. bassiana* ERL836 or JEF-007 was cultured on the 1/4SDA medium and adults of western flower thrips were exposed to the fungal mass for 3 days. Collected cuticle-interacting fungus was subjected to RNA extraction and differentially expressed genes were compared. In the DEG analysis using the JEF-007 sequencing reads (Supplementary Table S2), a total of 643 genes were up-regulated, and 982 genes were down-regulated when infecting western flower thrips (Fig. 4a). Approximately 1.5 times more contigs belonged to down-regulated genes than up-regulated genes. Most fold changes were in the range of − 2 to 2 and accounted for 80.9% of total DEGs (1625 DEGs). Among up-regulated genes (643 genes), 51% were assigned to the biological process category, 32% to the molecular function category, and 17% to the cellular component category. Among down-regulated genes (982 genes), 44% were assigned to the molecular function category, 41% to the biological process category, and 15% to the cellular component category. Both up- and down-regulated genes in the biological process category were involved in metabolic processes. Most up-regulated genes in the molecular function category had catalytic activity, whereas most down-regulated genes in this category were involved in binding. A similar number of contigs in most GO categories of the cellular component category were found among up-regulated genes, while genes with a membrane function were some of the most down-regulated genes.

In the DEG analysis using the ERL836 sequencing reads (Supplementary Table S2), a total of 1197 genes were up-regulated, and 360 genes were down-regulated (Fig. 4b). ERL836 treatment resulted in approximately twice more up-regulated contigs than down-regulated contigs. Most fold changes were in the range of–2 to 2 and accounted for 71.96% of total DEGs (2471 DEGs). Among up-regulated genes (1197 genes), 38.4% were in the biological process category, 43.1% in the molecular function category, and 18.5% in the cellular component category. Similarly, among down-regulated genes, 39.8% were in the biological process category, 40.0% in the molecular function category, and 20.1% in the cellular component category. Metabolic process-related genes were the most abundant up-regulated genes. Among genes with a molecular function, most contigs were annotated as encoding proteins with catalytic activity followed by binding and heterocyclic compound binding. Among cellular component genes, similar numbers of contigs were found in most of the GO categories.

Randomly selected up-regulated genes from the ERL836 and JEF-007 contigs were subjected to qRT-PCR for validation. The fold change levels of the selected genes as analyzed by qRT-PCR were consistent with the Illumina sequencing analysis results (Supplementary Figure S1).

### GO enrichment of shared DEGs between two isolates

**Fig. 5:**
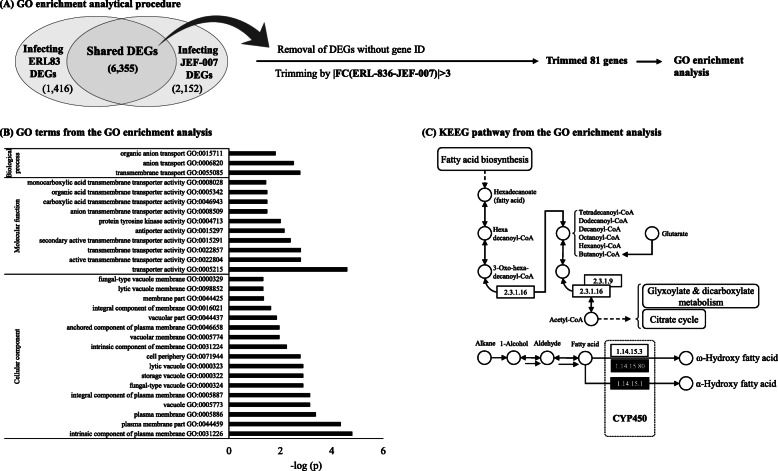
GO enrichment of shared DEGs between cuticle-interacting JEF-007 and ERL836. Shared DEG contigs between cuticle-interacting JEF-007 and infecting ERL836 that showed more than ±3 fold change difference were subjected to GO enrichment analysis using g:Profiler to investigate significant functional differences when infecting thrips (**a**). From the results of enrichment, significantly different GO terms (**b**) and KEGG pathway (**c**) were summarized. Dotted parts of the KEEG pathway can be involved with CYP450 (cytochrome P 450). The *B. bassiana* does not provide enough gene IDs for enrichment analysis, so alternatively *Saccharomyces cerevisiae* with much larger gene IDs was used as a reference and the *p*-value was corrected for multiple testing using the FDR procedure with a threshold of 0.05. Trimmed DEGs and enriched GO terms were provided in the Supplementary Table S3

A list of shared DEGs between cuticle-interacting *B. bassiana* ERL836 and JEF-007 was derived from RNA-seq data for enrichment analysis. A total 6355 DEGs were shared by the two *B. bassiana* isolates, but 3576 genes without gene IDs were excluded from the analysis (Fig. 5a). To investigate differences, 81 genes were trimmed based on the fold change value between infecting ERL836 and JEF-007 (**|**FC (ERL836-JEF-007)**|** > 3). From the GO enrichment analysis (Supplementary Table S3), 30 GO terms were significantly affected by shared DEG groups (FDR < 0.05) (Fig. 5b). Transporter activity (GO:0005215), plasma membrane part (GO: 0044459), and intrinsic component of plasma membrane (GO:0031226) functions were profoundly affected. According to pathway (KEGG) from the GO enrichment, the significantly affected pathway was the fatty acid degradation pathway (KEGG:00071) (−log(*p*) >  1.4), which is related to cytochrome P450 (Fig. 5c). Some *cytochrome P450* genes (*CYP* gene) in the shared gene groups showed different gene expression levels; highly up-regulated in ERL836 but down-regulated in JEF-007. The fold change value of *CYP539B1* in ERL836 was 2.34, while in JEF-007 it was − 0.73. The *CYP655C1* had fold change value of 3.87 in ERL836 but − 0.1 in JEF-007. Additionally, *CYP5099A1* was upregulated at 2.66-fold in ERL836, but downregulated at − 3.21-fold in JEF-007.

## Discussion

In this work, the two *B. bassiana* isolates, ERL836 and JEF-007 showed high similarities in biological features, such as morphology, conidial productivity and virulence against western flower thrips. Not only these two isolates, but also many other *B. bassiana* isolates look similar and are regarded to be similar active ingredient when being developed as biopesticides. The highly similar morphological characteristics of isolates can lead to potential conflicts when they are submitted to patent application and product development. So, in this work to further characterize two patented *B. bassiana* isolate ERL836 and JEF-007, their genome structures and gene expression patterns when infecting western flower thrips were analyzed and compared. The genome structures of the two isolates were very similar, but gene expression patterns were quite different when interacting with the cuticles of western flower thrips as the initial step of infection. Different transcriptional responses could therefore be used as a characteristic to differentiate the two *B. bassiana* isolates.

The two *B. bassiana* isolates, JEF-007 & ERL836 had similar genome structures, but their scaffold numbers were different from *B. bassiana* ARSEF2860, which might be due to the different sequencing technologies used to obtain the genomes of the isolates. JEF-007 and ERL836 isolates were sequenced using the recent PacBio sequencing technology which reads ca. 10 Kb at a time (maximum 20 Kb) followed by error correction, whereas the genome of ARSEF2860 was generated using Illumina pair-end sequencing technology, which reads short fragments of 100 bp. Ten and nine contigs of more than 1 Mb (considered the minimum size of chromosome) were generated for JEF-007 and ERL836, respectively. It is therefore possible that the two isolates have ca. 10 chromosomes, whereas ARSEF2860 has been reported to have six chromosomes [25]. The ERL836, JEF-007 and ARSEF2860 as a reference shared 8409 genes, but each of isolate had less than 30 unique ortholog genes, very small. Unique genes of each isolate were predicted to be involved in fungal life cycles including growth (FluG domain-containing protein, flocculin, methyltransferase, and protein kinase domain-containing protein), stress response (indoleamine-dioxygenase subfamily, tlh3, metallophosphoesterase, and transposase-like protein) and nutrient uptake (alpha/beta hydrolase fold-3 and ribose-phosphate pyrophosphokinase). The rest half of them is hypothetical protein gene.

For the RNA-sequencing, conidia were extracted from the cuticle at day three, when mortalities in the entire assay were around 50%, so some fungi were able to penetrate the cuticle, develop infection and kill the host. But some other conidia might have initial hyphal penetration to the cuticle or under delayed germination on the cuticle, probably due to host defense mechanism or other unfavorable environmental factors. Thus, it needs to be understood that the conidia analyzed in this RNA-sequencing might represent a combination of these several situations. In GO analysis of ERL-836 DEGs, there were significantly more up-regulated genes than down-regulated genes in various GO categories, when compared with JEF-007. Particularly highly up-regulated ERL836 GO terms were detected in the biological process and molecular function of ERL836. This result indicates that when interacting with the cuticle of thrips, possibly ERL836 much easily overcomes the defense mechanisms of thrips compared to JEF-007, but JEF-007 looks having difficulty in that initial step. In ERL836, many fungal genes were highly up-regulated in the infection process. For example, the eukaryotic aspartyl protease showed an 11.2-fold increase in ERL836 when interacting with the cuticle of western flower thrips. This aspartyl protease has a potential to degrade host insect tissues and disable anti-microbial peptides [26].

In the GO enrichment analysis, *S. cerevisiae* was used as a reference rather than *B. bassiana* because *Saccharomyces* GO IDs have been established much better than *B. bassiana* GO IDs and provide a good eukaryotic model system for genetic and biochemical mechanisms. However, no gene IDs were found for 3576 genes which were shared by both of ERL836 and JEF-007. This is a limitation of this GO enrichment analysis. In spite of this limitation, GO categories of membrane transporters were significantly different between infecting JEF-007 and ERL836. ABC transporters are membrane transporters that play an essential role in *B. bassiana* infection as well as in infection by the phytopathogenic fungi ABC1 and BcatrB [27]. Transporter capability depends on activation of antioxidative enzymes [28]. *B. bassiana* harbors 21 ABC transporter genes, but not all ABC transporters contribute to fungal pathogenicity [29].

In the enrichment analysis, fatty acid degradation pathway was significantly different between JEF-007 and ERL836. The *Cytochrome P450* genes have been found to be involved in fatty acid degradation. Interestingly, most *cytochrome P450* genes in cuticle-interacting ERL836 were up-regulated, but in JEF-007 they were down-regulated. This suggests that these *cytochrome P450* genes of ERL836 could play an important role in thrips infection in contrast to in JEF-007. In eukaryotic microorganisms, cytochrome P450 is involved in alkane oxidation. At least 83 genes encoding cytochrome p450s have been reported in *B. bassiana*, and a study of *B. bassiana* infection of silkworms revealed that cytochrome P450s are involved in infection and proliferation of *B. bassiana* [30]. In the previous study, CYP5099A1, CYP617A1, CYP617A2, CYP52G8, CYP539B1, and CYP655C1 were found to be involved in fatty acid degradation (KO00071), which is related to insect hydrocarbon degradation [31]. Cytochrome P450 enzymes can catalyze cascade formation of mono-oxidation products followed by diterminal oxidation and finally produce α-ω acids [32]. CYP505D4 is involved in sulfur metabolism (KO00920). CYP540B16, CYP61A1, CYP586B1, and CYP682H1 are involved in steroid biosynthesis (KO00100). The two *B. bassiana* isolates, JEF-007 and ERL836, shared the expression of CYP.

## Conclusions

In summary, the two *B. bassiana* isolates ERL836 and JEF-007 showed similar biological characteristics, such as morphology and virulence against western flower thrips as well as similar genome structure, but gene expression patterns were quite different when interacting with cuticle of western flower thrips. *B. bassiana* ERL836 appeared to interact with cuticle of thrips easily, while JEF-007 appeared to have more difficulty based on transcriptional analyses. Interestingly, in the enrichment analysis using shared DEGs between two interacting isolates, plasma membrane-mediated transporter activity and fatty acid degradation pathway including cytochrome P450 were more active in ERL836. This comparative approach using shared DEG analysis could be applied to easily characterize the difference of the two patented *B. bassiana* isolates, ERL836 and JEF-007.

## Methods

### Fungal isolates

*B. bassiana* ERL836 was obtained from the Entomology Research Laboratory (ERL) Worldwide Collection of Entomopathogenic Fungi of the University of Vermont, USA, and was originally collected from soil in California, USA. *B. bassiana* JEF-007 was obtained from the Insect Microbiology and Biotechnology Laboratory (IMBL), Chonbuk National University, Republic of Korea. Fungal isolates were grown on quarter strength Sabouraud dextrose agar (1/4SDA; Difco, USA) in the dark at 25 ± 1 °C and stored in 20% (v/v) glycerol at − 80 °C.

### Hyphal growth and conidial production

Fungal mycelia masses from 7-day-old *B. bassiana* isolates were collected into 1.5 ml Eppendorf tubes containing 0.03% (v/v) siloxane solution. After 30 s of shaking on a vortex mixer (Vortex-Genie 2TM; VWR Scientific, NY, USA), 2 ml of conidial suspension (1 × 10^7^ conidia ml^− 1^) was dropped on the middle of a ¼ SDA plate (90 mm diameter). Plates were kept in a 25 °C incubator and observed for 9 days under a stereoscopic microscope. Fungal spore productivity was evaluated using millet-based solid cultured granules. Fungal granules were produced by modification of the protocol of Kim et al [33]. Briefly, 200 g of millet was mixed with 100 ml of distilled water with 50% citric acid (160 μl) in a polyvinyl bag. Then the millet was autoclaved at 121 °C for 15 min and cooled down at room temperature. One milliliter of fungal conidial suspension (1 × 10^7^ conidia ml^− 1^) was inoculated and incubated at 25 ± 2 °C for 10 days. After drying the fungal granules till they had a moisture content of less than 10%, the 0.1 g millet-based medium was collected into 1.5 ml microtubes with 0.03% (v/v) siloxane solution (Silwet, FarmHannong, Seoul, Republic of Korea). The fungal suspension was then vortexed vigorously for 3 min and the number of conidia was counted using a hemocytometer (Cat No. 2960408, Marienfeld, Bad Mergentheim, Germany) under a microscope (400×).

### Bioassay against western flower thrips

To compare the virulence of the two *B. bassiana* JEF-007 and ERL836, a colony of western flower thrips was received from the National Institute of Horticultural and Herbal Science, Republic of Korea. Insects were placed on filter paper moistened with 2 ml of distilled water in a breeding dish (90 mm diameter, 50 mm height). Each stage of thrips was reared in a different breeding dish and provided with fresh bean sprouts every day. Old sprouts with eggs were transferred to new breeding dish every second day. Thrips were kept at 25 ± 2 °C, 40 ± 10% relative humidity, and a 14:10 (L:D) photoperiod. For fungal bioassay against the thrips, conidial suspensions were prepared and adjusted to 1 × 10^5^, 1 × 10^6^, 1 × 10^7^, and 1 × 10^8^ conidia ml^− 1^ using 0.03% (v/v) siloxane solution. A cucumber disc (diameter 60 mm) was placed on a filter paper in a Petri-dish (diameter 60 mm) and 1 ml of conidial suspension was sprayed on the cucumber disc and dried for 10 min at room temperature. Distilled water containing 0.03% Silwet (v/v) served as a non-treated control. To maintain high humidity, 100 μl distilled water was added to the dishes. Eleven two-day-old thrips adults were transferred to the fungus-treated dish (11 adults dish^− 1^) and coved with lids. Treated dishes were kept at 24 ± 2 °C and living adults were counted every day. Each treatment was replicated three times (3 plates per treatment). Lethal concentration 50 (LC_50_) values of the two isolates were calculated using a Probit analysis (SPSS Inc., 2018).

### Whole genome sequencing

For whole genome sequencing of *B. bassiana* ERL836 at Macrogen (www.macrogen.com; Macrogen Inc., Seoul, Korea), genomic DNA and RNA were extracted from 7-day-old fungal mycelia. DNA quantity was assessed using Pico-green staining (Invitrogen, Cat No. P7589) and Victor 3 fluorometry. To assess DNA quality, gel electrophoresis was performed. The concentration of genomic DNA (81.79 ng/μl) was measured using a Nano Drop spectrophotometer (Thermo Scientific) and a Qubit fluorometer (Life Technology). For PacBio RS sequencing, 8 g of input genomic DNA was used for 20 kb library preparation. For gDNA with a size range less than 17 Kb, a Bioanalyzer 2100 (Agilent) was used to determine the actual size distribution. Genomic DNA was sheared with g-TUBE (Covaris Inc., Woburn, MA, USA) and purified using AMPure PB magnetic beads (Beckman Coulter Inc., Brea, CA, USA) if the apparent size was greater than 40 kb. The gDNA concentration was measured using both a Nano Drop spectrophotometer and a Qubit fluorometer, and approximately 200 ng μl^− 1^ of gDNA was run on a field-inversion gel. Total of 10 μl of library was prepared using the PacBio DNA Template Prep Kit 1.0 (for 3 ~ 10 Kb). SMRT bell templates were annealed using PacBio DNA/Polymerase Binding Kit P6. PacBio DNA Sequencing Kit 4.0 and eight SMRT cells were used for sequencing. Subsequent steps were based on the PacBio Sample Net-Shared Protocol, which is available at http://pacificbiosciences.com/.

### Genome analysis

All the genome-level comparisons were outsourced to Macrogen except genome alignment and KEEG analysis. When *B. bassiana* JEF-007 and ERL836 genomes were compared using a Repeat Masker program (v4.0.5), another *B. bassiana* ARSEF2860 was used as a reference. To obtain fungal secreted protein proportions, TMHMM (v2.0) was used and the protein sequences of the *B. bassiana* isolates were subjected to ortholog analysis using OrthoMCL (v.2.0.3). Sequences encoding peptides shorter than 10 amino acids and those with more than 20% stop codons were removed prior to blastp analysis (v2.2.25+; E-value 1E-5). To compare the sequences of two *B. bassiana* isolates, the Nucmer (NUCleotide MUMmer) module of the MUMmer package was used and data were analyzed using the following three steps: maximal extract matching, match clustering, and alignment extension. Alignments of ERL836 with JEF-007, ERL836 with ARSEF2860, and JEF-007 with ARSEF2860 were analyzed by Assemblytics (http://assemblytics.com/) and dot blot figures were drawn. Lastly KEGG orthology (KO) was analyzed by the web-based server KAAS (http://www.genome.jp/tools/kaas/). For gene ortholog analysis and pathway mapping, query sequences of JEF-007, ERL836 and ARSEF2860 were inputted, respectively. Identification of orthologous genes was conducted by bi-directional best hit (BBH). A total of 348,165 ascomycete sequences (taken from the KEGG databases) were used as reference sequences.

### RNA extraction from thrips-infecting *B. bassiana* isolates

*B. bassiana* ERL836 or JEF-007 which was interacting with cuticle of western flower thrips adults was subjected to RNA extraction. Conidial suspension (70 μl, 1 × 10^7^ conidia ml^− 1^) of each isolate was spread on ¼SDA plate and cultured at 27 °C for 3 days. Then ca. 600 adults of western flower thrips were transferred to the cultured plate and the plate was incubated at 27 °C for 3 days. Infected thrips were collected into 1.5 ml microtubes with 0.03% (v/v) siloxane solution, and vortexed for 3 min. To exclude thrips bodies, the suspensions were filtered by sterilized iron mesh (ca. 80 μm^2^ pore size) and a fungal suspension was collected. As a control, fungal mass of each isolate was harvested from 6-day-old cultures on ¼SDA. The two different treatments were replicated three times (3 samples from the thrips-treated *B. bassiana* and another 3 samples from the *B. bassiana* only). Total RNAs were extracted by TRIzol reagent (Invitrogen Life Technologies, CA, USA) following the manufacturer’s instructions. RNA purity and integrity were quantified by ASP-2680 spectrophotometer (ACTGene, Piscataway, NJ, USA) and an Agilent Technologies 2100 Bioanalyzer (Agilent Technologies, Palo Alto, Cambridge, USA).

### RNA sequencing and analysis

Libraries of cuticle-interacting and cuticle-non-interacting ERL836 or JEF007 were made using the Truseq RNA kit (Illumina, San Diego, USA) following the manufacturer’s protocol at Macrogen. Multiple indexing adapters were ligated to the ends of the double-stranded cDNA and then enriched by PCR to create DNA library templates. In each isolate, cuticle-interacting and cuticle-non-interacting samples were sequenced in parallel using an Illumina HiSeq 2000 sequencer. Before analyzing the sequences, quality scores were checked by Fast QC (ver 0.11.7). After receiving sequencing raw data from Macrogen, the following analyses were conducted in our laboratory. For efficient and robust de novo reconstruction of transcriptomes, Trinity (ver 2.8.3) was used (https://github.com/trinityrnaseq/). To identify candidate coding regions within transcript sequences, Trans-Decoder (ver 5.5.0) was used. In the first step, long ORFs were extracted, and then likely coding regions were predicted. To cluster similar nucleotide sequences into clusters meeting a user-defined similarity threshold (0.9 in this analysis), CD-HIT-EST (version 4.7) was used. To quantify transcript abundances, Kallisto (ver 0.45.0) was used to build an index form from the fasta form of target sequences and non-infecting and infecting libraries were compared. Transcripts per million (TPM) of non-infecting and infecting samples was calculated. Raw signals were normalized using a log_2_-based transformation. Fold-change statistical tests were performed and log_2_|FC|≧2 was defined as statistically significant differential expression. Genes were blasted using the Blast2Go program. Analysis was conducted by local blast with *B. bassiana* ARSEF2860 as a reference sequence. Statistical significance threshold was 1.0E-10 and the number of blast hits was set to one. Gene ontology (GO) analysis of up- and down-regulated genes was performed using InterPro (online) in the Blast2Go program. The public EMBL-EBI database was used to scan sequences against InterPro’s signatures. Up- and down-regulated genes were annotated at GO level 2.

### Validation of RNA-sequencing

Cuticle-interacting and cuticle-non-interacting RNA samples were subjected to reverse transcription (RT) using AccuPower® RT PreMix (Bioneer, Daejeon, Republic of Korea) with the oligo (dT) 15 primer (Promega, MI, USA). A set of primers for RT-PCR (Supplementary Table S4) were designed at Snap Dragon (http://www.flyrnai.org/snapdragon). RT-PCR was performed as follows: an initial denaturation at 94 °C of 5 min followed by 34 cycles of 30 s at 94 °C, 30 s at (Tm)°C, and 30 s at 74 °C, followed by a final extension for 10 min at 74 °C (C-1000, Bio Rad, Hercules, CA, USA). To validate up-regulated genes, five genes of JEF-007 and three genes of ERL836 were randomly selected and subjected to quantitative RT-PCR (qRT-PCR). To remove gDNA contamination, RNA extracted by the Trizol method (described above) was treated with 1 μg of DNaseI (Invitrogen Life Technologies, CA, USA). cDNAs were generated using the AccuPowder® Rocketscript RT PreMix kit (BioNeer) and oligo (dT) primer (Promega), and they were used as the template for PCR amplification. Then qRT-PCR was performed using Thunderbird® Syber® qPCR mix (QPS-201, TOYOBO, Japan) on a 96-well Bio-Rad CFX96 Real-Time PCR System (Bio-Rad, USA). Cycling parameters for qRT-PCR were as follows: denaturation for 1 min at 95 °C, and then 40 cycles of 15 s at 95 °C, 1 min at 60 °C followed by melting with an increase in temperature of 0.5 °C per 5 s starting from 65 °C to 95 °C. Primers for *B. bassiana* actin (=γ-actin, GenBank Accession No: HQ232398) were used as an internal control to obtain relative expression levels [34]. △Ct (threshold cycle) was calculated as (Ct value of up-regulated genes) - (Ct value of *Bb*-actin) and subjected to the calculation of fold change value (2^-△△Ct^).

### GO enrichment analysis

Shared DEG contigs between JEF-007 and ERL836 which had more than 3-fold change difference were subjected to GO enrichment analysis to investigate the different mechanisms of the two fungal isolates in pathogenesis. In this analysis, blastn was performed with an E-value of 1.0E-10. Non-blasted and non-GO ID contigs were removed in this analysis. Functional enrichment was performed using the g:Profiler web server (http://bitt.cs.ut.ee/gprofiler/) [35]. Input query data list was matched with *Saccharomyces cerevisiae* (https://flybase.org/). The *B. bassiana* does not provide enough gene IDs for enrichment analysis, so alternatively *S. cerevisiae* with much larger analyzed gene IDs was used as a reference. The related *p*-value was corrected for multiple testing using the Benjamini-Hochberg False Discovery Rate (FDR) procedure with a threshold of 0.05.

### Data analysis

Data on the numbers of conidia, percentage of live thrips and expression level in qRT-PCR were arc-sine transformed and analyzed using an ANOVA or generalized linear model (GLM) followed by Tukey’s honestly significant difference (HSD) for multiple comparisons. All the analyses were conducted using SPSS (SPSS Inc., 2018) at the 0.05 (α) level of significance.

## Supplementary Information

**Additional file 1 **: **Table S1.** de novo assembly of *B. bassiana* JEF-007 (A) and ERL846 (B) after the sequencing of whole genomes using Pac Bio RSII technology with error correction. **Table S2.** de novo assembly of *B. bassiana* RNA-sequencing raw data. **Table S3.** GO enrichment analysis of differentially expressed and shared genes between cuticle-interacting *B. bassiana* JEF-007 and ERL836. **Table S4.** Primers used in qRT-PCR for validation of *B. bassiana* RNA-sequencing. **Figure S1.** Validation of RNA-sequencing of *B. bassiana* ERL836 and JEF-007 using qRT-PCR.

## Data Availability

The genome of *B. bassiana* ERL836 has been deposited in the GenBank database under the project accession no. PPTI00000000.

## Abbreviations

1/4SDA: Quarter strength Sabouraud dextrose agar
ANOVA: Analysis of variance
DEG: Differentially expressed gene
FC: Fold change
FDR: False discovery rate
GLM: Generalized linear model
GO: Gene ontology
HSD: Honestly significant difference
KEEG: Kyoto encyclopedia of genes and genomes
LC_50_: Lethal concentration 50%
LT_50_: Lethal time 50%
