# Supplementary material for: Beauveria bassiana ERL836 and JEF-007 with similar virulence show different gene expression when interacting with cuticles of western flower thrips, Frankniella occidentalis
Source: BMC Genomics. 2020 Nov 27;21:836. doi: 10.1186/s12864-020-07253-y (PMC7694944; doi:10.1186/s12864-020-07253-y)
Supplement: Supplementary file 1 — Additional file 1 : Table S1. de novo assembly of B. bassiana JEF-007 (A) and ERL846 (B) after the sequencing of whole genomes using Pac Bio RSII technology with error correction. Table S2. de novo assembly of B. bassiana RNA-sequencing raw data. Table S3. GO enrichment analysis of differentially expressed and shared genes between cuticle-interacting B. bassiana JEF-007 and ERL836. Table S4. Primers used in qRT-PCR for validation of B. bassiana RNA-sequencing. Figure S1. Validation of RNA-sequencing of B. bassiana ERL836 and JEF-007 using qRT-PCR. [file 12864_2020_7253_MOESM1_ESM.pdf]

Supplementary Table S1. *de novo* assembly of *B. bassiana* JEF-007 (A) and ERL846 (B) after the sequencing of whole genomes using Pac Bio RSII technology with error correction

(A) *B. bassiana* JEF-007 WGS

Contig	Length (bp)	GC %	Depth
Contig 1	4,836,080	47.8	104
Contig 2	4,313,377	47.2	104
Contig 3	3,721,617	48.5	104
Contig 4	3,539,716	50.2	107
Contig 5	3,115,233	46.7	102
Contig 6	2,580,292	48.6	105
Contig 7	2,356,961	50.4	107
Contig 8	2,191,280	43.9	102
Contig 9	1,987,760	50.8	107
Contig 10	1,591,247	48.7	107
Contig 11	977,447	51.2	109
Contig 12	957,547	52.2	107
Contig 13	681,700	48.1	106
Contig 14	677,945	49.6	106
Contig 15	561,348	45.9	105
Contig 16	508,980	47	101
Contig 17	390,418	50.3	109
Contig 18	260,236	26.7	87
Contig 19	246,642	44.2	91
Contig 20	225,477	46.4	107
Contig 21	178,896	42.3	97
Contig 22	105,676	44.8	100
Contig 23	100,976	53.4	114
.			
.			
Contig 39	5,751	21	1
Total	36,538,394	48.04	105

The genome data of JEF-007 was generated from our previous work (Lee et al., 2018, Scientific Report).

Supplementary Table S1. Continued

(B) ***B. bassiana*** **ERL836 WGS**

Contig	Length (bp)	GC %	Depth
Contig 1	6,378,951	48.3	80
Contig 2	6,291,768	49.4	79
Contig 3	4,840,633	49.5	79
Contig 4	3,988,868	48.8	80
Contig 5	3,742,282	50.6	82
Contig 6	3,708,924	51.6	78
Contig 7	2,509,591	50.9	91
Contig 8	2,432,845	44.8	82
Contig 9	1,458,486	51.3	79
Contig 10	38,942	27	1,666
Contig 11	38,806	21.3	22
Contig 12	22,558	52.3	19
Contig 13	21,869	51.3	51
Contig 14	8,146	50.2	4
Contig 15	5,773	56	15
Total	35,488,442	49.33	82

Supplementary Table S2. *de novo* assembly of *B. bassiana* RNA-sequencing raw data

Category	ERL836		JEF-007	
	Non-infecting	Infecting	Non-infecting	Infecting
**Total trinity contig**	**29,407**	**76,109**	**30,372**	**143,934**
N25	2,473	1,778	7,705	3,901
N50	3,798	772	5,074	1,808
N75	2,410	377	3,238	599
GC (%)	53.33	49.87	53.26	53.48
**Total trimmed contig**	**9,602**	**32,416**	**8,658**	**54,596**
N25	2,796	1,275	2,793	1,617
N50	1,788	708	1,824	924
N75	1,152	435	1,221	495
GC (%)	56.38	49.7	56.5	55.17

In each isolate, cuticle-interacting fungal sample was prepared using 3-day old fungus which was treated to the adults of thrips. Non-interacting fugus was prepared using 6-day old culture on SDAY/4 medium. A threshold of 0.9 was applied to the trimming of the contigs.

Supplementary Table S3. GO enrichment analysis of differentially expressed and shared genes between cuticle-interacting *B. bassiana* JEF-007 and ERL836.

**Source (level 1)**	**Go term (level 2)**	**-$\log_{10}$(adjusted p)**	**Intersection (Yeast as reference)**
Cellular component	Intrinsic component of plasma membrane GO:0031226	4.785149535	YKR105C,YKL046C,YJL212C,YMR215W,YDL210W,YPL154C,YGL114W,YLR092W,YGR055W,YBR296C
	Plasma membrane part GO:0044459	4.33744124	YKR105C,YLL028W,YKL046C,YJL212C,YMR215W,YDL210W,YPL154C,YGL114W,YLR092W,YGR055W,YBR296C
	Plasma membrane GO:0005886	3.366235569	YKR105C,YOL002C,YLL028W,YKL046C,YJL212C,YJL145W,YDR536W,YMR215W,YDL210W,YPL154C,YGL114W,YCR028C,YOR328W,YLR092W,YLR004C,YGR055W,YBR296C,YGR213C
	Vacuole GO:0005773	3.137934389	YMR088C,YKR105C,YLL048C,YLL028W,YCL057W,YHL035C,YDL210W,YPL154C,YCR023C,YBR161W,YLR299W,YER065C,YGR213C
	Integral component of plasma membrane GO:0005887	3.137934389	YKR105C,YJL212C,YDL210W,YGL114W,YLR092W,YGR055W,YBR296C
	Fungal-type vacuole GO:0000324	2.874610043	YMR088C,YLL048C,YLL028W,YCL057W,YHL035C,YDL210W,YPL154C,YCR023C,YLR299W,YGR213C
	Storage vacuole GO:0000322	2.874610043	YMR088C,YLL048C,YLL028W,YCL057W,YHL035C,YDL210W,YPL154C,YCR023C,YLR299W,YGR213C
	Lytic vacuole GO:0000323	2.874610043	YMR088C,YLL048C,YLL028W,YCL057W,YHL035C,YDL210W,YPL154C,YCR023C,YLR299W,YGR213C
	Cell periphery GO:0071944	2.7687304	YKR105C,YOL002C,YLL028W,YKL046C,YJL212C,YJL145W,YDR536W,YMR215W,YDL210W,YPL154C,YDR150W,YGL114W,YCR028C,YOR328W,YBR162C,YLR092W,YLR004C,YGR055W,YBR296C,YGR213C

	Intrinsic component of membrane GO:0031224	2.238584979	YMR088C,YKR105C,YJR126C,YOL002C,YLL048C,YHL028W,YLL028W,YKL046C,YKL140W,YJL212C,YIL166C,YDR536W,YHL035C,YMR215W,YDL210W,YPL154C,YCR023C,YGL114W,YBR161W,YLR299W,YDR338C,YCR028C,YOR328W,YGL203C,YMR266W,YKL201C,YLR092W,YLR004C,YGL225W,YGR055W,YBR296C,YGR213C
	Vacuolar membrane GO:0005774	1.952699435	YMR088C,YKR105C,YLL048C,YLL028W,YHL035C,YDL210W,YCR023C,YBR161W,YLR299W
	Anchored component of plasma membrane GO:0046658	1.952699435	YKL046C,YMR215W,YPL154C
	Vacuolar part GO:0044437	1.850225548	YMR088C,YKR105C,YLL048C,YLL028W,YHL035C,YDL210W,YCR023C,YBR161W,YLR299W
	Integral component of membrane GO:0016021	1.623469558	YMR088C,YKR105C,YJR126C,YOL002C,YLL048C,YHL028W,YLL028W,YKL140W,YJL212C,YIL166C,YDR536W,YHL035C,YDL210W,YCR023C,YGL114W,YBR161W,YLR299W,YDR338C,YCR028C,YOR328W,YGL203C,YMR266W,YKL201C,YLR092W,YLR004C,YGL225W,YGR055W,YBR296C,YGR213C
	Membrane part GO:0044425	1.346345475	YMR088C,YKR105C,YJR126C,YOL002C,YLL048C,YHL028W,YLL028W,YKL046C,YKL140W,YJL212C,YIL166C,YJL145W,YDR536W,YHL035C,YMR215W,YDL210W,YPL154C,YCR023C,YGL114W,YBR161W,YLR299W,YDR338C,YCR028C,YOR328W,YGL203C,YMR266W,YKL201C,YLR092W,YLR004C,YGL225W,YGR055W,YBR296C,YGR213C
	Lytic vacuole membrane GO:0098852	1.32428122	YMR088C,YLL048C,YLL028W,YHL035C,YDL210W,YCR023C
	Fungal-type vacuole membrane GO:0000329	1.32428122	YMR088C,YLL048C,YLL028W,YHL035C,YDL210W,YCR023C

Molecular function	Transporter activity GO:0005215	4.591412798	YMR088C,YKR105C,YLL048C,YHL028W,YLL028W,YJL212C,YIL166C,YJL145W,YDR536W,YHL035C,YDL210W,YCR023C,YGL114W,YDR338C,YCR028C,YOR328W,YLR380W,YLR092W,YLR004C,YGL225W,YGR055W,YBR296C
	Active transmembrane transporter activity GO:0022804	2.787905169	YLL048C,YLL028W,YJL212C,YDR536W,YHL035C,YDL210W,YDR338C,YOR328W,YLR092W,YGR055W,YBR296C
	Transmembrane transporter activity GO:0022857	2.787905169	YMR088C,YKR105C,YLL048C,YLL028W,YJL212C,YIL166C,YDR536W,YHL035C,YDL210W,YGL114W,YDR338C,YCR028C,YOR328W,YLR092W,YGL225W,YGR055W,YBR296C
	Secondary active transmembrane transporter activity GO:0015291	2.384085096	YLL028W,YJL212C,YDR536W,YDL210W,YDR338C,YLR092W,YGR055W,YBR296C
	Antiporter activity GO:0015297	2.14690372	YLL028W,YDL210W,YDR338C,YLR092W,YGR055W
	Protein tyrosine kinase activity GO:0004713	1.996809448	YJL095W,YPL153C,YJL187C
	Anion transmembrane transporter activity GO:0008509	1.473089303	YMR088C,YLL048C,YIL166C,YDL210W,YCR028C,YLR092W,YGR055W
	Carboxylic acid transmembrane transporter activity GO:0046943	1.473089303	YMR088C,YLL048C,YDL210W,YCR028C,YLR092W,YGR055W
	Organic acid transmembrane transporter activity GO:0005342	1.473089303	YMR088C,YLL048C,YDL210W,YCR028C,YLR092W,YGR055W
	Monocarboxylic acid transmembrane transporter GO:0008028	1.434611546	YLL048C,YDL210W,YCR028C

Biological process	Transmembrane transport GO:0055085	2.756077078	YMR088C,YKR105C,YLL048C,YLL028W,YJL212C,YIL166C,YDR536W,YHL035C,YDL210W,YCR023C,YGL114W,YDR338C,YCR028C,YOR328W,YLR092W,YLR004C,YGL225W,YGR055W,YBR296C
	Anion transport GO:0006820	2.509257903	YMR088C,YKR105C,YLL048C,YIL166C,YJL145W,YHL035C,YDL210W,YCR028C,YLR380W,YLR092W,YGR055W,YBR296C
	Organic anion transport GO:0015711	1.806813286	YMR088C,YKR105C,YLL048C,YJL145W,YHL035C,YDL210W,YCR028C,YLR380W,YLR092W,YGR055W

Supplementary Table S4. Primers used in qRT-PCR for validation of *B. bassiana* RNA-sequencing

Gene		Sequences (5′ - 3′)	Annotation	GenBank Accession No.
Bb γ-actin	F	GTCAAGTCATCACCATTGGC	γ -actin	HQ232398
	R	CGTAGAGATCCTTGCGAACA		
ERL836-4090	F	ATGTCCTTTGAGGAGCGAAA	Alcohol dehydrogenase I	XM_008601263.1
	R	TCTACAAGGGCCTCAAGGAG		
ERL836-4178	F	GATGACGGCCATGAGAGAAT	ABC-2 type transporter	XM_008596878.1
	R	TTCGTTGGCTTCGTCTTCTT		
ERL836-8766	F	CGGATGTCGGCATTTACTTT	Kynureninase-like protein	XM_008595984.1
	R	CATGATGACAATCTCGCTCG		
JEF007-1692	F	TGAACACCGAGGAGGAAATC	ABC transporter	XM_008596200.1
	R	GGAATTGTCCCAGCAAAAGA		
JEF007-1871	F	AACAAGACGGGTTCCTGTTG	ABC transporter	XM_008603257.1
	R	CGACTAGACCCGACACCATT		
JEF007-2735	F	CTCGGCTACTGGTTCTACCG	Heme peroxidase	XM_008603121.1
	R	TTTCCTTGTCATAGGTGCCC		
JEF007-6217	F	ATATCGAGCAGGATGTTGCC	LCCL domain-containing protein	XM_008600272.1
	R	TCATCAGTCGCACGCTTTAC		
JEF007-6893	F	GCTCACAGTTTCGCCTTTTC	ABC-2 type transporter	XM_008596200.1
	R	CCGGCAAGATAGGTGTTTGT		

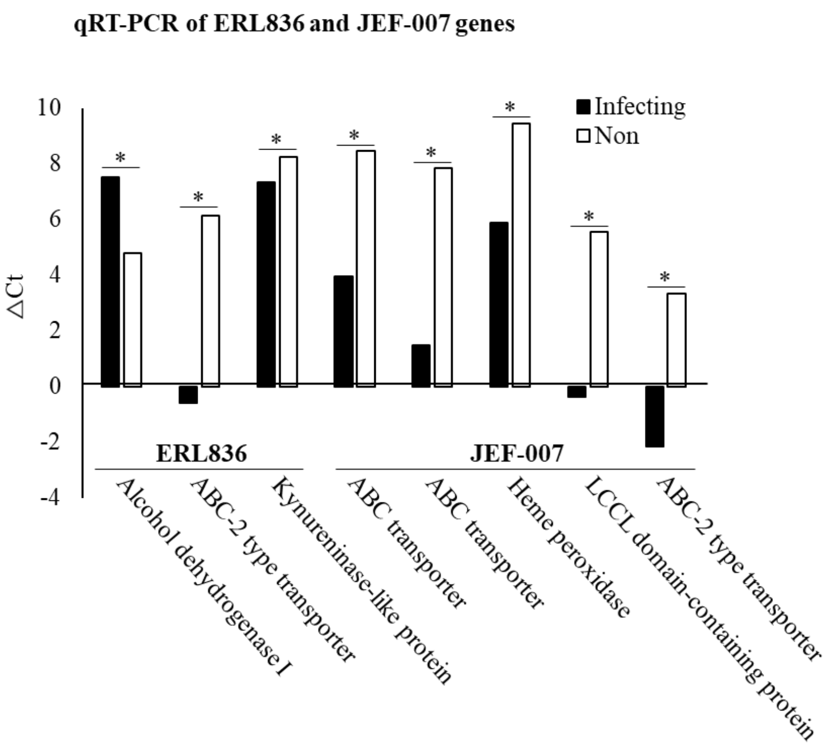

Supplementary Figure S1. Validation of RNA-sequencing of *B. bassiana* ERL836 and JEF-007 using qRT-PCR. For the qRT-PCR of up-regulated genes, primers were established (**Supplementary Table S4**), and after the qRT-PCR, ΔCq values were analyzed based on the Ct value of *B. bassiana* actin gene. *B. bassiana* actin (= γ -actin, GenBank accession number: HQ232398) primers were used as an internal control to calculate relative expression levels. Asterisk (*) indicates that the two values are significantly different (p<0.05).
